# Performance Habits: A Framework Proposal

**DOI:** 10.3389/fpsyg.2020.01815

**Published:** 2020-08-19

**Authors:** Sylvain Laborde, Daniela Kauschke, Thomas J. Hosang, Florian Javelle, Emma Mosley

**Affiliations:** ^1^Department of Performance Psychology, German Sport University Cologne, Cologne, Germany; ^2^Normandie Université, Caen, France; ^3^Universität Mannheim, Mannheim, Germany; ^4^Helmut Schmidt University, Hamburg, Germany; ^5^Department of Sport Medicine, German Sport University Cologne, Cologne, Germany; ^6^Southampton Solent University, Southampton, United Kingdom

**Keywords:** optimization, automatic processes, habits, performance, human

“*We are what we repeatedly do. Excellence, then, is not an act but a habit.”*

Aristotle

## Introduction

Improving one's performance is a persistent desire for many individuals. Performance is often used as an umbrella term to describe the behavior and activities of individuals or larger entities, such as organizations, and could ultimately be linked to evolutionary success (Buss, 2019). Specifically, we define performance as how effectively an action is executed and how successful behavior is to achieve a goal [based on (McGarry, 2013; Raab et al., 2015)].

One common tip to improve performance is developing habits. Various definitions of habits coexist in the literature, and in this paper we consider habits as processes “by which a stimulus generates an impulse to act as a result of a learned stimulus–response association” [(Gardner, 2015), p. 277]. Habits are driven by automatic processes in that they do not require conscious and intentional processing of related information (Graybiel, 2008; Lally and Gardner, 2013). Habits should be distinguished from routines, which can be viewed as organized activities with purpose, direction, sequence, outcomes, and repetition, but which are not necessarily based on a learned stimulus–response association (Clark, 2000; Charmaz, 2002; Gardner, 2015). Rituals are often used as a synonym for routines; however routines are seen as being rather mostly instrumental to reach a goal, while rituals encompass a symbolic meaning in a specific group context (Fiese et al., 2002). Habitual behavior can be distinguished in terms of habitual instigation—habitually “deciding” to do something—and habitual execution—habitually “doing” something (Gardner et al., 2016, 2020). Regarding habit formation, Lally and Gardner (2013) identified four basic stages: First, a decision must be made to take action. Second, the decision to act must be translated into action. Third, the behavior must be repeated, and finally, the new action must be repeated in a way that leads to automaticity.

The notion that habits can lead to performance optimization is widespread. In fact, a search for “performance habits” returns more than 500 million hits on Google (with mostly business websites, such as Forbes, “10 Daily Habits of the Most Productive Leaders”^1^). More than 3,000 books are available on Amazon, with some bestsellers such as “High Performance Habits: How Extraordinary People Become That Way” (Burchard, 2017) or “Tools of Titans: the Tactics, Routines, and Habits of Billionaires, Icons, and World-Class Performers” (Ferriss, 2017). The majority of non-scientific “expert” recommendations on habits to improve performance originate from those who have reached an elite level in a specific domain (e.g., business, sports, music, politics, etc.) or a certain social status. Subsequently they then present those habits as key influential factors for their success (Burchard, 2017; Ferriss, 2017). However, beyond those anecdotal reports and popular interest, to date very few scientific researchers investigated the effectiveness of habits to improve one's performance. So far, the main themes in habit research in human focuses on the formation of health habits (Lally and Gardner, 2013; Gardner, 2015), which is of course of utmost individual and societal importance. Habit research in the field of human performance however is sparse—with some exceptions regarding academic, cognitive, and athletic performance (Cotrena et al., 2016; Dubuc et al., 2019; Longo et al., 2019; Fiorella, 2020; Kristo et al., 2020)—and we aim in this opinion piece to outline a range of dimensions which could benefit from the conceptualization of habits for optimizing performance.

## Performance Habits

We refer to performance habits as habits targeting performance optimization, considering performance as defined above. The theoretical characteristics of habits make them very relevant for performance (Gardner, 2015; Wood, 2017). Indeed paying attention to the numerous factors influencing performance can be quite effortful and cognitively demanding (Raab et al., 2015). Habits, reflecting actions relying on automatic functioning, free up resources for further top-down processing (Graybiel, 2008; Lally and Gardner, 2013) and reduce motivational impairments (Stojanovic et al., 2020). At this point, we should distinguish between habits and the automatic processes driving skill automaticity observed in expert performance, given in this case that the automatic processes are not necessarily the result of a learned stimulus–response association but rather the result of an extensive learning/training phase that automatized skills via a modification in brain activation patterns (Baker and Young, 2014; Yang, 2015). Nonetheless, habits can provide a basis to optimize learning/training in facilitating its instigation and execution (Gardner et al., 2016, 2020), ultimately facilitating skill automatization. In a nutshell, habits can be seen as a tool to help in transferring human behavior driven by conscious processes to human behavior driven by unconscious processes, therefore optimizing resources and performance. Altogether these characteristics make habits an important mechanism through which people can self-regulate and achieve long-term goals (Wood, 2017). Consequently, people willing to achieve performance goals would benefit from integrating performance habits to their preparation in order to optimize it.

In the following discussion, we introduce a list of dimensions related to activities that have been linked to performance improvement and that could also form into performance habits. To establish this list of dimensions representing the basis of the performance habits framework, general principles for mapping reviews were followed (Miake-Lye et al., 2016). Mapping reviews are particularly useful when there is a large diversity of research as a first step to a systematic review and to identify gaps within an area (Cooper, 2016; Perryman, 2016). The database search^2^ was directed to identify meta-analyses and reviews concerning factors influencing human performance, with the aim to derive higher-order dimensions of strategies improving performance that can be turned into habits.

**Figure 1:**
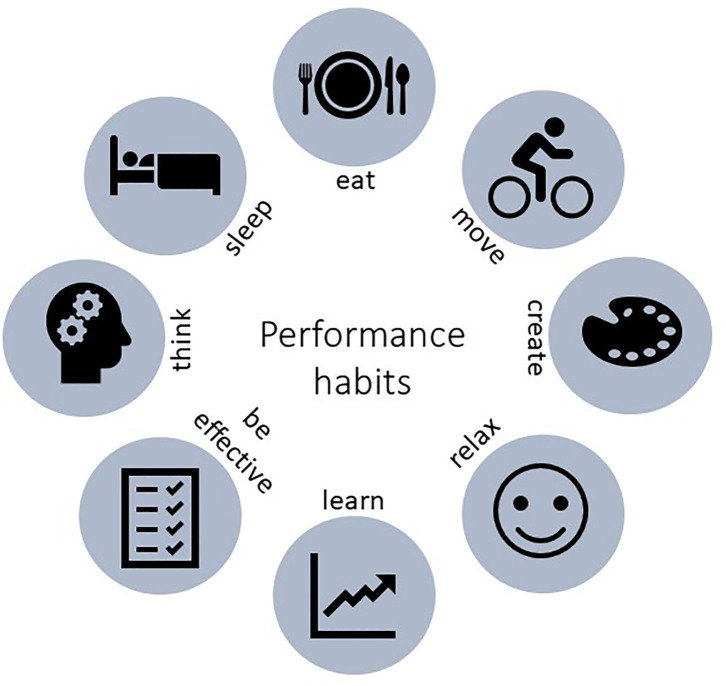
The eight performance habit dimensions are summarized into action verbs: eat (dietary behavior), move (exercise), sleep (sleep), relax (psychological well-being), learn (strategies to foster learning), productivity (strategies to foster productivity), think (strategies to foster executive functioning), and create (strategies to foster creativity). We are aware that the action verbs do not reflect accurately the full spectrum of the dimensions considered, however we believe that they are helpful in order to showcase how individuals may take action to transform them into performance habits.

Eight main dimensions emerged from the visual map of the mapping review. First, we find the classical three dimensions identified by neuroscience to underpin performance (Briguglio et al., 2020): eat (i.e., dietary behaviors), move (i.e., exercise), and sleep. Furthermore, we outline five additional dimensions: psychological well-being, as well as strategies to foster learning, productivity, executive function, and creativity. Those eight dimensions are summarized in Figure 1.

### Performance Habit Dimensions

#### Dietary Behavior

Dietary behaviors have a high impact on performance, from academic performance at school to athletes' performance (Burke et al., 2004; Rampersaud et al., 2005). Dietary behavior habits can be related to overall diet, such as the Mediterranean (Soltani et al., 2019), vegetarian (Craddock et al., 2019; Viguiliouk et al., 2019), vegan (Lopez et al., 2019), or gluten-free (Taetzsch et al., 2018) diet. Dietary behavior habits may also be related to supplementation (Van De Walle and Vukovich, 2018; Clifford et al., 2019; Wilson-Barnes et al., 2020) or to having no meal at all, for example, skipping breakfast (Rampersaud et al., 2005), or with intermittent fasting (Aird et al., 2018; Cho et al., 2019).

#### Exercise

Regular exercise sustains performance in many domains and across the lifespan (Heyn et al., 2004; Smith et al., 2010; Chang et al., 2012; Álvarez-Bueno et al., 2020). It is now widely acknowledged that exercise benefits from the conceptualization of habits (Hagger, 2019, 2020; Gardner et al., 2020; Phillips, 2020). Regarding performance, the type of exercise (e.g., aerobic, resistance, high-intensity interval training, stretching) should be considered according to the performance outputs expected (Wen et al., 2019; Lee and Stone, 2020; Martland et al., 2020; Wilke et al., 2020).

#### Sleep

Sleep is essential for performance, given that partial or chronic sleep deprivation may affect performance in many domains (Pilcher and Huffcutt, 1996; Dewald et al., 2010; Lee et al., 2020). The usefulness of habits for sleep interventions has been showcased by Rebar et al. (2020). Sleep-based interventions have been shown to be effective in triggering positive sleep habits (Dewald et al., 2010; Chung et al., 2017; Bonnar et al., 2018; Friedrich and Schlarb, 2018). Among the sleep habits promoted by sleep-based interventions, we find, for example, bedtime routines involving decreasing smartphone use (Shin et al., 2017), using relaxation methods (Laborde et al., 2019), or writing to-do lists (Scullin et al., 2018).

#### Psychological Well-Being

Psychological well-being (Ryff and Singer, 1996; Ryan and Deci, 2001; Boehm and Kubzansky, 2012) has been shown to positively influence academic performance (Amholt et al., 2020), work performance (Parker et al., 2003; Ford et al., 2011), and sport performance (Lundqvist, 2011). Among the interventions that have been used to promote psychological well-being (Bolier et al., 2013; Ryff, 2014), several methods could be turned into habits to improve performance, such as meditation (Tang, 2014; Chan et al., 2019), progressive muscle relaxation and autogenic training (Manzoni et al., 2008), slow-paced breathing (Zaccaro et al., 2018), journaling (Hensley and Munn, 2020), nature exposure (Kaplan and Berman, 2010), or expressing gratitude (Wood et al., 2010).

#### Strategies to Foster Learning

Beyond the mere amount of practice/time spent to learn, the characteristics of learning (e.g., new material, new skill) are very likely to impact subsequent performance in many domains like in academia (Dunlosky et al., 2013), work (Salas and Cannon-Bowers, 2001), and sport (Macnamara et al., 2016). If the habits to learn will depend on the specific learning object, some recommendations can be made at a meta-level, such as developing habits regarding (1) learning techniques, for example, massed vs. distributed learning (Cepeda et al., 2006), (2), learning environment (Dunlosky et al., 2013), considering both the physical (e.g., library vs. at home) and the social aspects (e.g., with a teacher, with peers, alone), and finally (3) learning material, such as books, podcasts, videos, Smartphone apps, etc. (Koçak et al., 2016; Delgado et al., 2018).

#### Strategies to Foster Productivity

Productivity refers to the optimization of personal workflow and effectiveness (Fosse et al., 2015; Lewis et al., 2019). It is recommended that individuals develop a personal workflow management system (Lackey et al., 2014), with habits underlying productivity, such as distraction minimization (Lewis et al., 2019) and time management interventions (Fosse et al., 2015; Lewis et al., 2019). Distraction minimization (Lewis et al., 2019) would involve, for example, developing habits to effectively handle phone calls, emails, or any internet-based/social network distractions. Time optimization (Fosse et al., 2015; Lewis et al., 2019) is achieved through the programming of short breaks, with efficient note reporting and classification techniques (Lewis et al., 2019), and with using speed-reading techniques (Rayner et al., 2016).

#### Strategies to Foster Executive Functioning

Regardless of the performance domain considered, performance to reach a goal will rely on executive functioning, the part of cognitive functioning that allows us to perform goal-directed behavior (Diamond, 2013). Cognitive training programs have been developed to target individuals across the lifespan and have been shown to be effective from pre-schoolers (Scionti et al., 2019) to older adults (Lampit et al., 2014). The extent to which executive functions can be trained or the cognitive training modality of performance would transfer to other domains of performance is still debated (Jak et al., 2013; Simons et al., 2016); most of the research show that improvements are related to the cognitive task or domain-trained (Melby-Lervåg and Hulme, 2013; Butler et al., 2018). Therefore, determining the most relevant executive functions for the performance domain would determine the type of habit developed.

#### Strategies to Foster Creativity

In some domains, performance would be related to doing something novel, to synthesize and combine in a new way existing information, thus requiring creativity, also referred to as divergent thinking (Dietrich and Kanso, 2010; Raab et al., 2015). Creativity training focuses on the development of cognitive skills and the heuristics involved in skill application and should use realistic exercises appropriate to the domain at hand (Scott et al., 2004; Valgeirsdottir and Onarheim, 2017). Social interactions should be encouraged to allow brainstorming with other people (Al-Samarraie and Hurmuzan, 2018). Finally, the contexts fostering creativity should be clarified, for example, exercising (Frith et al., 2019) or listening to music (Ritter and Ferguson, 2017).

## Conclusion

The aim of this opinion paper was to suggest a framework to investigate habits targeting performance optimization, going beyond the existing health-focused habit research. We developed the idea of performance habits which can be split into individual strategies to improve the following dimensions: eat, move, sleep, relax, learn, be effective, think, and create.

Following the purpose of mapping reviews (Cooper, 2016; Miake-Lye et al., 2016; Perryman, 2016), the goal of this opinion paper was not to reach a definitive conclusion about the field of performance habits but rather to showcase this large area. It is hoped that it will establish the ground for future systematic investigation and experimental endeavor. Consequently, the framework presented should not be considered as definitive but as subject to evolution. The dimensions that we suggest here need to be further refined, with the help of quantitative and qualitative research, to understand the extent to which they effectively contribute to performance according to the domain considered. If some have already received attention from habit researchers (e.g., dietary behavior, physical activity, sleep), we argue that the other dimensions would benefit as well from the theoretical consideration of habits. Additionally, research should also consider to which extent the habit–performance relationship can be seen as a “one-size-fits-all” association or should be better individualized (e.g., Sales et al., 2014) and also investigate the influence of potential moderators such as the use of pharmaceuticals (Marcora, 2016) and biological rhythms (Atkinson and Reilly, 1996). Furthermore, the investigation of these eight dimensions raises some methodological concerns. For example, logging habits linked to strategies fostering learning, productivity, executive functions, and creativity may differ from what has been done so far in habit research and potentially require the development of specific instruments. It should also be taken into account that each dimension may interact with the others regarding their influence on performance. Finally, one should consider that striving for performance may also include the reversal of bad habits, which otherwise may eventually lead to addictive/compulsive behavior (Malloy-Diniz et al., 2019).

At the theoretical level, there is a need to investigate whether habits, based on their characteristics (Gardner, 2015; Wood, 2017), do serve performance better than repetitive (but non-habitual) ways of realizing certain actions. This will help to develop our understanding of the role of habits in helping individuals to successfully achieve their goals, with the ultimate objective of exploring the extent to which habits are linked to evolutionary human success in that they help to offer stability in an everchanging, complex, and modern human environment (Buss, 2019; Furley, 2019).

## Author Contributions

SL and DK prepared the first draft. TH, FJ, and EM provided critical comments to significantly improve the manuscript. All authors contributed to the article and approved the submitted version.

## Conflict of Interest

The authors declare that the research was conducted in the absence of any commercial or financial relationships that could be construed as a potential conflict of interest.

## Footnotes

^1^Retrieved on June 15, 2020: https://www.forbes.com/sites/deeppatel/2017/07/14/10-daily-habits-of-the-most-productive-leaders/#49599b407e9c.

^2^The search was completed on January 13, 2020, with the Web of Science, all databases included, for the past 10 years (period 2010–2020). The following keywords were entered: meta-analysis or review in the Title field and performance in the All Fields field. All document types (full papers, proceedings papers, meeting abstract, etc.) in English language were considered. All Web of Science categories were included, and 34,654 unique results were retrieved. The results were exported to the reference software Zotero. From the titles and abstracts screening, SL and DK extracted higher-order themes matching the intended scope of the framework (i.e., strategies to improve performance that can be turned into habits) and represented them on a visual map. The visual map was then refined with TH, FJ, and EM until consensus was reached.
